# Application of Multiple Imputation for Missing Values in Three-Way Three-Mode Multi-Environment Trial Data

**DOI:** 10.1371/journal.pone.0144370

**Published:** 2015-12-21

**Authors:** Ting Tian, Geoffrey J. McLachlan, Mark J. Dieters, Kaye E. Basford

**Affiliations:** 1 School of Agriculture and Food Science, the University of Queensland, Brisbane, Queensland, Australia; 2 School of Mathematics and Physics, the University of Queensland, Brisbane, Queensland, Australia; University of California, Berkeley, UNITED STATES

## Abstract

It is a common occurrence in plant breeding programs to observe missing values in three-way three-mode multi-environment trial (MET) data. We proposed modifications of models for estimating missing observations for these data arrays, and developed a novel approach in terms of hierarchical clustering. Multiple imputation (MI) was used in four ways, multiple agglomerative hierarchical clustering, normal distribution model, normal regression model, and predictive mean match. The later three models used both Bayesian analysis and non-Bayesian analysis, while the first approach used a clustering procedure with randomly selected attributes and assigned real values from the nearest neighbour to the one with missing observations. Different proportions of data entries in six complete datasets were randomly selected to be missing and the MI methods were compared based on the efficiency and accuracy of estimating those values. The results indicated that the models using Bayesian analysis had slightly higher accuracy of estimation performance than those using non-Bayesian analysis but they were more time-consuming. However, the novel approach of multiple agglomerative hierarchical clustering demonstrated the overall best performances.

## Introduction

Multi-way data analysis has become common in many areas of research involving multivariate data. Three-way three-mode pattern analysis refers to the combined use of such clustering and ordination procedures. Its application to multivariate multi-environment trial (MET) data has provided a comprehensive summary of the patterns of variation and the interactions among the three modes, genotypes, environments and attributes, for plant breeders and other scientists interested in plant improvement [1, 2]. However, many multivariate MET datasets are incomplete and the presence of missing values cause complications because most analytical methods developed for multivariate data assume complete data arrays [3, 4]. This is the case for (iterative) clustering and ordination procedures where the inability to routinely apply them to incomplete datasets has been an obstacle to their wider usage (as a full data array is needed to provide starting values for any necessary iteration). Thus, it is important to obtain the best possible estimates of missing values to form a complete multi-way MET data array which can then be subjected to multi-way pattern analysis.

There are some statistical methods and mathematical algorithms specifically designed to handle incomplete two-way two-mode data matrices. In one of them, multiple imputation (MI) [5, 6] is used to generate different imputed values for each missing value to form different complete datasets. Then the different complete two-way datasets were analysed in order to obtain estimates of the parameters of the corresponding models because these parameters were the main interest for some authors [7]. These different complete datasets were defined as the “estimated data arrays” as they were the complete data arrays containing the estimated missing values using MI approaches.

While we wanted to use multiple imputation to generate different imputed values for each missing cell (and eventually obtain one estimated data array for each incomplete multivariate MET dataset), the estimation of the (different) parameters in the various models used in the imputation process were not of concern to us. Thus, we focused on using different MI approaches to obtain “good” estimates of the missing values to form a complete “estimated data array” which could then be analysed by three-way three-mode pattern analysis, rather than for parameter estimation. The MI methods mentioned above (for two-way two-mode data matrices) were modified to take into account the three-way structure of multivariate MET data. We also introduced one novel MI approach which does not have an underlying model that can be written in a similar format to the others.

To demonstrate the use of MI for estimating missing values in multivariate MET data, two real complete MET datasets and four simulated complete MET datasets were considered. Missing values were generated by randomly deleting values in the full datasets. The methods were assessed by comparing the original complete data arrays with the “estimated data arrays”, i.e., the complete data arrays containing estimated missing values. This enabled us to compare all of our methods for imputing missing values. Again, we stress that this was more important to us than the relative efficiency of the various estimators for the parameters in the models used in some of the imputation methods.

Some brief notation about the three-way three-mode data structure is described in the Materials and Methods. The basic algorithms for various MI approaches and corresponding modification in terms of multivariate MET datasets are also described. We then present the six multivariate MET datasets and the random generation of missing values, followed by the results of comparing the original complete data arrays with the complete data arrays containing estimated missing values. We end by discussing the implications of our findings.

## Materials and Methods

### Three-way three-mode MET data

**Fig 1:**
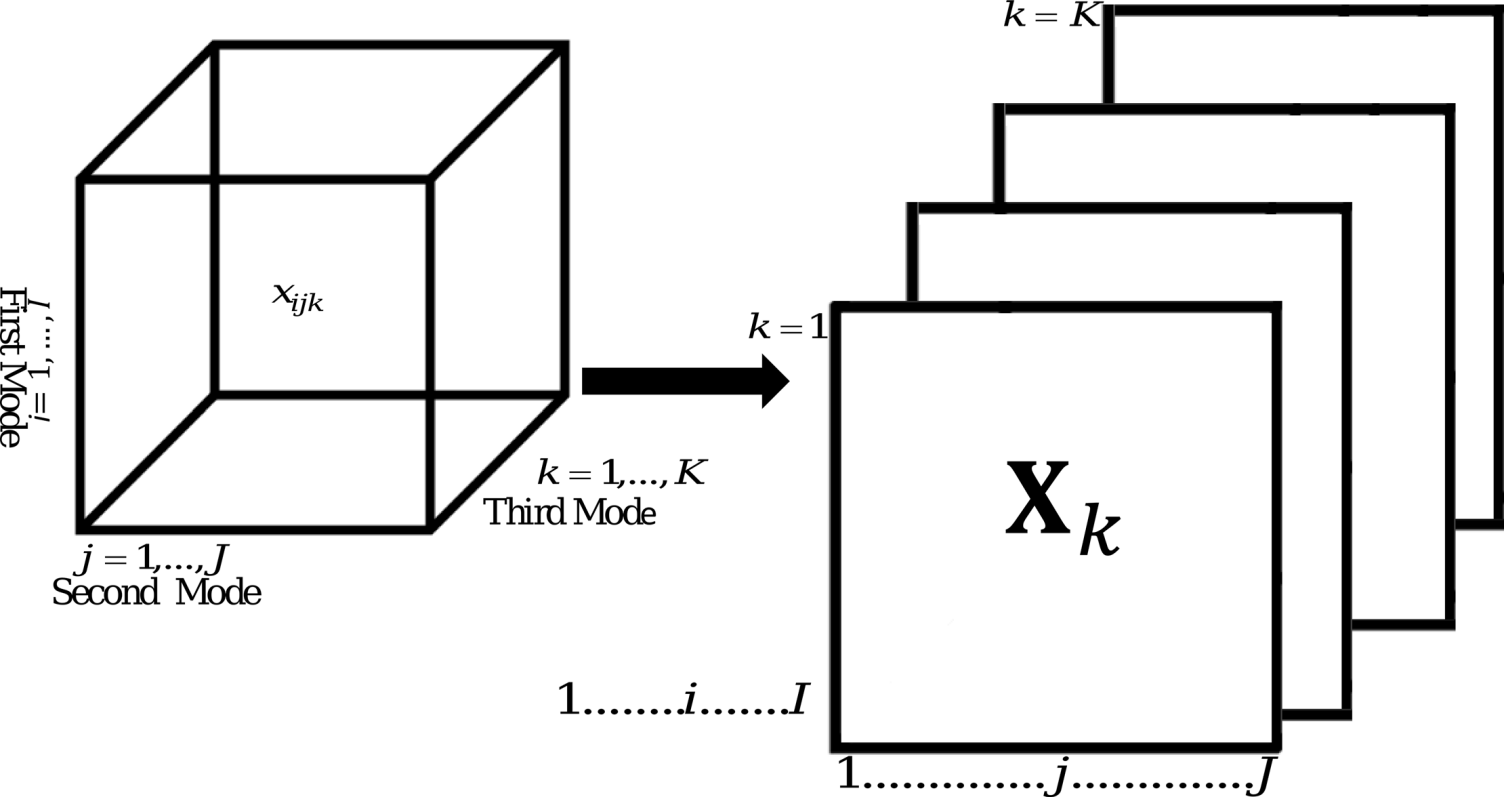
Three-way three-mode MET data array.

A MET data array generally consists of *I* genotypes, *J* environments and *K* attributes. It can be written as a collection of frontal slices **X**_*k*_ (*I*×*J* matrix, *k* = 1,…,*K*), where rows are genotypes and columns are environments, and each cell x_*ijk*_ is the value measured on the *i* ^*th*^ genotype in the *j* ^*th*^ environment for the *k* ^*th*^ attribute [8, 9] (Fig 1).

Each frontal slice **X**_*k*_ corresponds to *I* genotype responses across *J* environments for a particular attribute, such as yield, moisture or test weight. Each attribute has its own measurement units. There are two types of vectors for each frontal slice, a row vector $x_{ik}^{\prime }$ (i.e. measurements on the *i* ^*th*^ genotype for the *k* ^*th*^ attribute for all *J* environments) and a column vector **x**_*jk*_ (i.e. all *I* genotype responses measured in the *j* ^*th*^ environment for the *k* ^*th*^ attribute). We conducted column standardization in order to remove the environmental main effects, but retain the correlation among attributes (over genotypes) for each environment [1, 10, 11], i.e. we standardized each column vector **x**_*jk*_ prior to the analysis. It can be defined as:

$${\tilde{x}}_{ijk}=\frac{x_{ijk}-{\bar{x}}_{.jk}}{s_{jk}}$$

$$s_{jk}=\sqrt{\frac{\sum_{i=1}^{I}{\left(x_{ijk}-{\bar{x}}_{.jk}\right)}^{2}}{I-1}}$$

where the environment main effect is removed as $\sum_{i}{\tilde{x}}_{ijk}=0$ and the correlation among attributes for each environment is retained as:

$$Corr_{{\text{x}}_{jk},{\text{x}}_{jk^{\prime }}}=Corr_{{\tilde{\text{x}}}_{jk},{\tilde{\text{x}}}_{jk^{\prime }}}.$$

**Fig 2:**
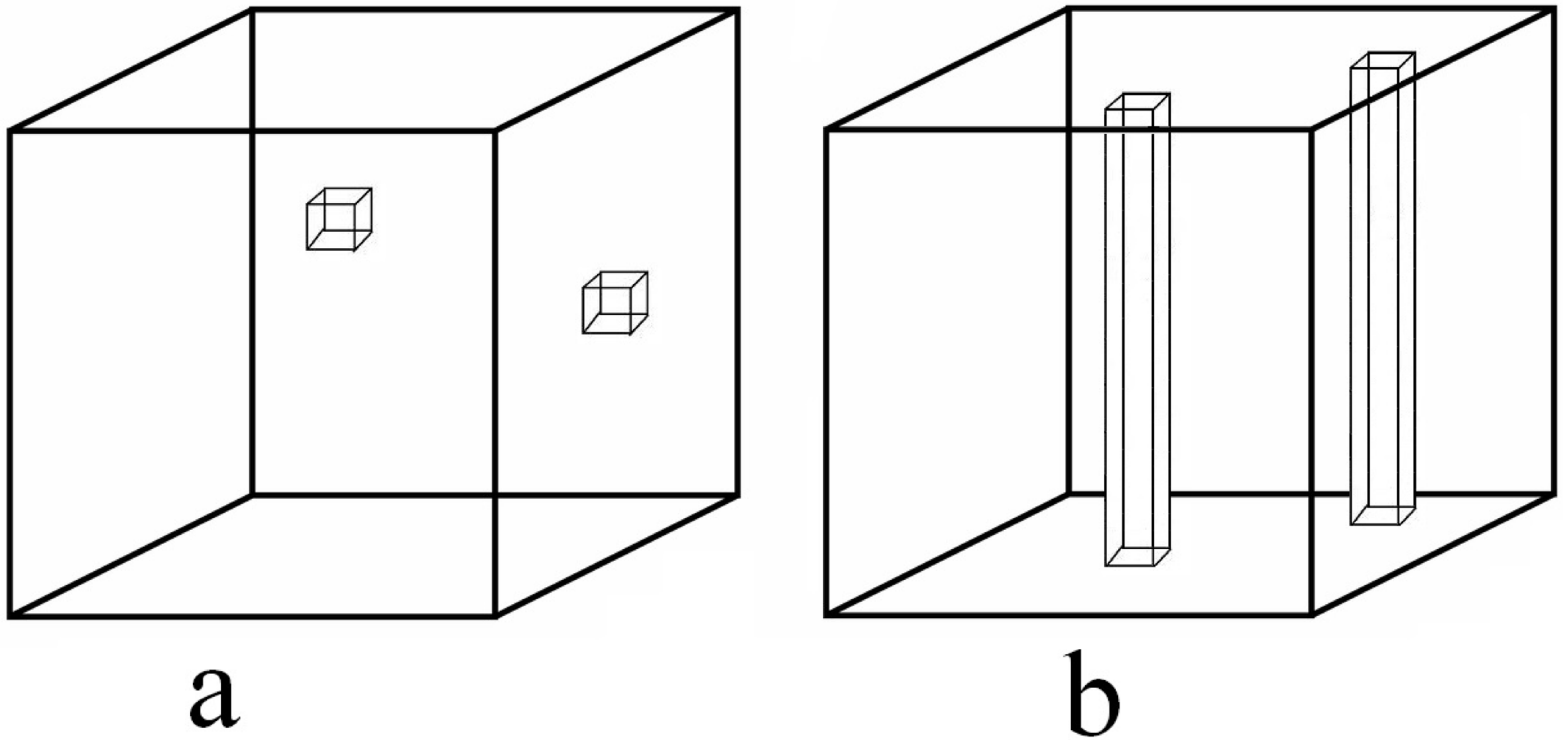
Patterns of missing values in our three-way MET data array. (a) missing cells, (b) missing columns.

Kroonenberg [9] discussed the various types of missing values in three-way three-mode data, and they are described here in terms of multivariate MET data:

- Single observations missing, e.g., individual genotypes in particular environments for a specific attribute are missing. These are missing cells in the three-way three-mode array (Fig 2A).
- Column missing, e.g., a particular attribute is not measured on any genotype in a particular environment. This would correspond to a missing column (**x**_*jk*_) in our three-way array (Fig 2B) and is quite common. A missing row ($x_{ik}^{\prime }$) where a particular attribute is not measured in any environment for a particular genotype is extremely rare in practice and will not be considered here.

Initially, we rearranged the standardized multivariate MET data array by writing it as a two-way wide matrix ${\tilde{X}}_{I\times JK}$, where the *I* rows are the *I* genotypes and *JK* columns are the *J* environments nested within each of the *K* attributes. It can be viewed as the following matrix including missing values:

$${\tilde{X}}_{I\times (JK)}=\left[\begin{array}{ccccccccccccccccc} {\tilde{x}}_{111} & \cdots & {\tilde{x}}_{1j1} & \cdots & {\tilde{x}}_{1J1} & .... & {\tilde{x}}_{11k} & \cdots & {\tilde{x}}_{1jk} & \cdots & NA & .... & {\tilde{x}}_{11K} & \cdots & {\tilde{x}}_{1jK} & \cdots & {\tilde{x}}_{1JK}\\ \vdots & & & & NA & & \vdots & & NA & & NA & & & & NA & & \\ NA & & & & \vdots & & NA & & \vdots & & NA & & & & \vdots & & \\ {\tilde{x}}_{I11} & \cdots & {\tilde{x}}_{Ij1} & \cdots & {\tilde{x}}_{IJ1} & .... & {\tilde{x}}_{I1k} & \cdots & {\tilde{x}}_{Ijk} & \cdots & NA & .... & {\tilde{x}}_{I1K} & \cdots & {\tilde{x}}_{IjK} & \cdots & {\tilde{x}}_{IJK} \end{array}\right]$$

where *NA* indicates that the observation is not available (missing). Note that there are only missing cells and a missing column (corresponding to a particular attribute not being measured on any genotype in that particular environment), where we only consider one missing column as an example of missing columns.

### Multiple imputation approaches

We are interested in two patterns of missing values in the two-way wide matrix ${\tilde{X}}_{I\times JK}$ of the multivariate MET data, i.e. missing cells (e.g. a data value missing for a particular attribute for a particular genotype in a particular environment) and missing columns (e.g. data values missing for a particular attribute for all genotypes in a particular environment). We consider the different MI approaches in terms of how they take into account these missing patterns when: (1) the estimation task is the same for missing cells and columns; (2) the estimation task is different for missing cells and columns. Under (1), we study imputation approaches based on multiple agglomerative hierarchical clustering (MAHC) and the normal distribution model (NORM) [6]. Under (2), we study imputation approaches based on the normal regression model (NRM) [3] and predictive mean matching (PMM) [12–14].

The latter three common MI approaches (NORM, NRM and PMM) were modified in terms of the multivariate MET data structure. In each of these, Bayesian analysis under Gibbs sampling [15] and non-Bayesian analysis were used in implementing the estimation task.

### Multiple Agglomerative Hierarchical Clustering (MAHC)

When investigating the genotype response pattern in multivariate MET data, it is most common to cluster the genotypes in terms of the measurements on the attributes in each of the environments. If there were only missing cells, then we could use an agglomerative hierarchical clustering procedure on the two-way wide standardized matrix ${\tilde{X}}_{I\times JK}$ to provide an estimate of a missing value for a particular genotype (by using the attribute value within the same environment of the genotype (or the genotype group) with which the genotype with the missing value first merged in the agglomerative process). However, if an attribute is not measured on any genotype in a particular environment, we cannot do this. We were not able to devise any procedure for estimating missing values by clustering genotypes when a whole column of the ${\tilde{X}}_{I\times JK}$ matrix was missing.

It was decided to rearrange the ${\tilde{X}}_{I\times JK}$ matrix as an ${\tilde{X}}_{J\times IK}$ matrix and cluster the environments. Then even when an attribute was not measured on any genotype within a particular environment, there would be no missing columns in the matrix. Hence we could use agglomerative hierarchical clustering of the environments to replace missing values for a particular attribute for all genotypes in a particular environment by the values of the same attribute for each genotype within the environment (or environment group) with which the environment with missing values first merged. This process of clustering environments to estimate missing values can also be used to estimate missing cells, i.e., when there was a missing value for a particular attribute for a particular genotype for a particular environment.

The process of estimating missing values using agglomerative hierarchical clustering of the environments, as described in the previous paragraph, only provided a single estimate for each missing value. We wanted a multiple imputation approach. In achieve this, we chose different subsets (or combinations) of attributes for each imputation of hierarchical agglomerative clustering of the environments, as that would result in (potentially) different hierarchies of the environments and subsequent estimates of missing values.

We initially tried to implement this MI procedure by choosing *K*_*h*_ attributes (by a uniform sample from the *K* measured attributes) for imputation *h*, *h* = 1,…,*H*, (*H* being the total number of imputations) with the value of *K*_*h*_ being any value from 1 to *K*. We conducted an agglomerative hierarchical clustering of the environments using the measurements of these *K*_*h*_ attributes on the *I* genotypes in the *J* environments expressed in the form of a *J*×*IK*_*h*_ two-way wide array. The environment response for a missing genotype-attribute value was estimated by the non-missing genotype-attribute value in the environment (or environment group) to which the environment with the missing value first joined. However, there was the possibility of not being able to estimate particular missing values because there were missing values on an attribute which was not chosen in that specific *K*_*h*_ attributes.

Hence we modified our MI procedure to ensure that all attributes were chosen in each imputation *h*. Thus for each *h*, we decided to choose all attributes (as in agglomerative hierarchical clustering) as well as *K*_*h*_ attributes with the value of *K*_*h*_ being any of the values from 1 to *K*-1. We conducted one agglomerative hierarchical clustering of the environments using all of the *K* attributes (with data in the form of a *J*×*IK* two-way wide matrix) and another agglomerative hierarchical clustering of the environments using the measurements of these *K*_*h*_ attributes (with data in the form of a *J*×*IK*_*h*_ two-way wide array). In each case, the environment response for a missing genotype-attribute value was estimated by the non-missing genotype-attribute value in the environment (or environment group) to which the environment with the missing value first joined. If two estimates were obtained for a particular missing value, they were averaged. This process was repeated *H* times, and the final estimates of missing values were the averages of the corresponding estimated values from each of the *H* imputations.

This multiple agglomerative hierarchical clustering used a dissimilarity measure between environments (and environment groups) based on squared Euclidean distance and a grouping strategy which minimized incremental sum of squares [16]. It takes into account different combinations of attributes at each imputation, leading to average values (over the imputations) of the estimated missing values for each cell.

### Normal Distribution Model (NORM)

Let ${\tilde{x}}_{ik}$ be the *J*×1 vector corresponding to the (standardized) responses for the *i* ^*th*^ genotype grown in all *J* environments for attribute *k*. The values are taken from the two-way wide standardized matrix ${\tilde{X}}_{I\times JK}$ and would have been part of the *i* ^*th*^ row in that matrix. Then it is assumed that this vector, ${\tilde{x}}_{ik}$ is multivariate normally distributed with mean vector **μ**_*ik*_ (a *J*×1 vector) and covariance matrix $\sum_{{}_{ik}}=\sigma_{ik}^{2}\;I$ (a *J*×*J* matrix), with **I** denoting an Identity matrix of size *J*×*J*. The corresponding mean vector **μ**_*ik*_ and covariance matrix **Σ**_*ik*_ are unknown. This could be written as:

$${\tilde{x}}_{ik}|\mu_{ik},\Sigma_{ik}∼N(\mu_{ik},\Sigma_{ik}).$$

We obtain the estimated values of unknown parameters **μ**_*ik*_ and **Σ**_*ik*_ using Bayesian analysis and non-Bayesian analysis as follows.

To conduct the Bayesian analysis, we needed to construct the prior distributions of parameters **μ**_*ik*_ and **Σ**_*ik*_. Here, we assumed that there was no strong prior information, so the Jeffrey’s prior distribution [17] would be as follows:

$$f(\mu_{ik},\sigma_{ik}^{2})=\frac{1}{\sigma_{ik}^{2}}$$

$${\tilde{x}}_{ik}|\mu_{ik},\sigma_{ik}^{2}∼N(\mu_{ik},\sigma_{ik}^{2}I),i=1,\ldots ,I.$$

As a constant value (here $\frac{1}{\sigma_{ik}^{2}}$) is not a very realistic probability density function, it is an improper prior distribution for $f(\mu_{ik},\sigma_{ik}^{2})$. However, when applying Bayes’ rule, this constant value prior distribution leads to a proper posterior probability density function, introducing some information about **μ**_*ik*_ and $\sigma_{ik}^{2}$ [17]. Then the posterior distribution was derived by

$$f(\mu_{ik},\sigma_{ik}^{2}|{\tilde{x}}_{ik})={\left(2\pi \sigma_{ik}^{2}\right)}^{-J/2}\exp\left\{-\frac{1}{2}\frac{\sum {\left({\tilde{x}}_{ik}-\mu_{ik}\right)}^{T}({\tilde{x}}_{ik}-\mu_{ik})}{\sigma_{ik}^{2}}\right\}\frac{1}{\sigma_{ik}^{2}}.$$

Then the full conditionals of $f(\sigma_{ik}^{2}|{\tilde{x}}_{ik},\mu_{ik})$ and $f(\mu_{ik}|{\tilde{x}}_{ik},\sigma_{ik}^{2})$ were:

$$\begin{array}{l} f(\sigma_{ik}^{2}|{\tilde{x}}_{ik},\mu_{ik})\propto f(\sigma_{ik}^{2},\mu_{ik}|{\tilde{x}}_{ik})\\ \;={\left(2\pi \right)}^{-J/2}{\left(\sigma_{ik}^{2}\right)}^{-(\frac{J}{2}+1)}\exp\{-\frac{1}{2\sigma_{ik}^{2}}\sum {\left({\tilde{x}}_{ik}-\mu_{ik}\right)}^{T}({\tilde{x}}_{ik}-\mu_{ik})\}\\ \;={\left(2\pi \right)}^{-J/2}{\left(\sigma_{ik}^{2}\right)}^{-(\frac{J}{2}+1)}\exp\left\{-\frac{JV_{\mu }^{k}}{2\sigma_{ik}^{2}}\right\} \end{array}$$

where $V_{\mu }^{k}=\frac{\sum {\left({\tilde{x}}_{ik}-\mu_{ik}\right)}^{T}({\tilde{x}}_{ik}-\mu_{ik})}{J}$ is the sample covariance matrix for known **μ**_*ik*_, and

$$\begin{array}{l} f(\mu_{ik}|{\tilde{x}}_{ik},\sigma_{ik}^{2})\propto f(\mu_{ik},\sigma_{ik}^{2}|{\tilde{x}}_{ik})\\ \;\propto \exp[-\frac{1}{2\sigma_{ik}^{2}}\sum {\left({\tilde{x}}_{ik}-\mu_{ik}\right)}^{T}({\tilde{x}}_{ik}-\mu_{ik})]\\ \;\propto \exp\{-\frac{1}{2\sigma_{ik}^{2}}(J\mu_{ik}^{\text{T}}\mu_{ik}+\sum {\tilde{x}}_{ik}^{\prime }{\tilde{x}}_{ik}-2\mu_{ik}\sum {\tilde{x}}_{ik})\}\\ \;\propto \exp\left\{-\frac{\mu_{ik}^{\text{T}}\mu_{ik}\;\text{-}\;2\mu_{ik}\sum {\tilde{x}}_{ik}/J}{2\sigma_{ik}^{2}/J}\right\}\\ \;\propto \exp\left\{-\frac{{\left(\mu_{ik}-{\bar{x}}_{k}\right)}^{T}(\mu_{ik}-{\bar{x}}_{k})}{2\sigma_{ik}^{2}/J}\right\} \end{array}$$

where ${\bar{x}}_{k}=\sum {\tilde{x}}_{ik}/J$ is the sample mean vector. Therefore, $\sigma_{ik}^{2}∼\mathrm{IG}(J/2,JV_{\mu }^{k}/2)$ and $\mu_{ik}\sim Ν({\bar{x}}_{k},\frac{\sigma_{ik}^{2}}{J})$. The posterior distributions of parameters **μ**_*ik*_ and $\sigma_{ik}^{2}$ were the normal distribution and inverse-gamma distribution, respectively.

Using Gibbs sampling [15], the estimates of the parameters **μ**_*ik*_ and $\sigma_{ik}^{2}$ were obtained. Gibbs sampling is Markov Chain Monte Carlo (MCMC) methodology. It was used to generate Z = (Z_1_,Z_2_,…,Z_*n*_) from a target probability density function (*pdf*) *f*(*z*), given the conditional *pdf f*(*z*_*i*_ | *z*_1_,*z*_2_,…*z*_*i*−1_,*z*_*i*+1_,…,*z*_*n*_). During the process of Gibbs sampling, the Markov Chain was generated from a sequence of conditional distributions.

From the above derivation, the conditional posterior distributions of parameters **μ**_*ik*_ and $\sigma_{ik}^{2}$ were the normal and inverse-gamma distribution, respectively. After the full distributions of parameters **μ**_*ik*_ and $\sigma_{ik}^{2}$ were obtained, the estimates of **μ**_*ik*_ and $\sigma_{ik}^{2}$ were obtained by generating a large number of extracted samples (here 5500) from those distributions and setting the estimate equal to their expectation values, i.e.

$${\hat{\mu }}_{ik}=E(\mu_{ik}),$$

$${\hat{\sigma }}_{ik}^{2}=E(\sigma_{ik}^{2}).$$

To conduct the non-Bayesian analysis, we also found the log-likelihood function to obtain maximum likelihood estimators (MLEs) for parameters **μ**_*ik*_ and $\sigma_{ik}^{2}$. The log-likelihood function for ${\tilde{x}}_{ik}$ was:

$$l(\mu_{ik},\sigma_{ik}^{2};{\tilde{x}}_{ik})=-(J/2)\log(2\pi \sigma_{ik}^{2})-\frac{1}{2}\sum \frac{{\left({\tilde{x}}_{ik}-\mu_{ik}\right)}^{T}({\tilde{x}}_{ik}-\mu_{ik})}{\sigma_{ik}^{2}}.$$

Thus, the MLEs for parameters **μ**_*ik*_ and $\sigma_{ik}^{2}$ were:

$${\hat{\mu }}_{ik}=\sum x_{ik}/J$$

$${\hat{\sigma }}_{ik}^{2}=\frac{1}{J}\sum {\left({\tilde{x}}_{ik}-{\hat{\mu }}_{ik}\right)}^{T}({\tilde{x}}_{ik}-{\hat{\mu }}_{ik}).$$

Therefore, the missing values ${\tilde{x}}_{ik}^{m}$ for the *i* ^*th*^ genotype measured on *J* environments for each attribute *k* could be drawn from the following equation:

$${\left({\hat{\tilde{x}}}_{ik}^{m}\right)}^{T}={\hat{\mu }}_{ik}+z{\hat{\sigma }}_{ik}$$ (1)

where **z** is random standard Normal vector. As a result, *H* imputed values for each missing values could be obtained by implementing the above process *H* times.

### Normal Regression Model (NRM)

Let $I_{jk}^{m}<I$ be the number of missing genotypes measurements in the *j* ^*th*^ environment for the *k* ^*th*^ attribute. Under the NRM, the particular standardised column vector (an *I*×1 vector) ${\tilde{x}}_{jk}$ containing $I_{jk}^{m}$ missing values can be expressed as a partition into two vectors, one containing non-missing values ${\tilde{x}}_{jk}^{obs}$ (an $(I-I_{jk}^{m})\times 1$ vector), and the other containing missing values ${\tilde{x}}_{jk}^{m}$ (an $I_{jk}^{m}\times 1$ vector). The response (or measurement) for one attribute *k* measured on a particular genotype *i* in a particular environment *j* is not independent of the responses for the other attributes measured on that genotype in that environment. Hence, the column vectors (genotype responses) for attribute *k* in environment *j* are not independent of the column vectors (genotype responses) for the other attributes *k*′ (*k*′ = 1,…,*K*, *k*′≠*k*) in that environment.

We assumed a standardized column vector ${\tilde{x}}_{jk}$ satisfied the regression model ${\tilde{x}}_{jk}∼N(X^{*}\beta_{jk},\sigma_{jk}^{2}I)$, where **β**_*jk*_ (a *N*×1vector) is the usual regression coefficient, **I** is an *I*×*I* identity matrix, and **X** ^*^ is the *I*×*N* design matrix containing elements from the *I* genotype responses for the *N* columns in the two-way wide matrix.

In order to modify the models used with two-way data arrays to take account of the multivariate nature of the measurements on each genotype in each environment, we employed the design matrix **X** ^*^ which could be expressed in four ways (i.e. there were four options for the *N* columns). In these options, the “zero” elements in matrix **X** ^*^ (or $X_{m}^{*}$ defined shortly) substituted for missing values. Thus, the missing values do not contribute to the normal regression model. However, there were a few “true” zero values in the data array because of the column standardization conducted prior to the analysis. These zero values do not contribute to the model either, as their original values corresponded to the average environment effect.

The four ways to express the design matrix **X** ^*^ were as follows:

1. The first option contained elements from the *I* genotype responses over the (*JK*-1) columns, i.e. *I*×(*JK*-1) matrix.
  $$\left[\begin{array}{ccccccccccccccccc} {\tilde{x}}_{111} & \cdots & {\tilde{x}}_{1j1} & \cdots & {\tilde{x}}_{1J1} & .... & {\tilde{x}}_{11k} & \cdots & * & \cdots & 0 & .... & {\tilde{x}}_{11K} & \cdots & {\tilde{x}}_{1jK} & \cdots & {\tilde{x}}_{1JK}\\ \vdots & & & & 0 & & \vdots & & * & & 0 & & & & 0 & & \\ 0 & & & & \vdots & & 0 & & * & & 0 & & & & \vdots & & \\ {\tilde{x}}_{I11} & \cdots & {\tilde{x}}_{Ij1} & \cdots & {\tilde{x}}_{IJ1} & .... & {\tilde{x}}_{I1k} & \cdots & * & \cdots & 0 & .... & {\tilde{x}}_{I1K} & \cdots & {\tilde{x}}_{IjK} & \cdots & {\tilde{x}}_{IJK} \end{array}\right]$$
  where the column corresponding to the particular environment *j* for particular attribute *k* was discarded, “*” denotes the discarded column
2. The second option contained elements from the *I* genotype responses over the (*J*-1) environments for each attribute, i.e. an *I*×*K*(*J*-1) matrix.
  $$\left[\begin{array}{ccccccccccccccccc} {\tilde{x}}_{111} & \cdots & * & \cdots & {\tilde{x}}_{1J1} & .... & {\tilde{x}}_{11k} & \cdots & * & \cdots & 0 & .... & {\tilde{x}}_{11K} & \cdots & * & \cdots & {\tilde{x}}_{1JK}\\ \vdots & & * & & 0 & & \vdots & & * & & 0 & & & & * & & \\ 0 & & * & & \vdots & & 0 & & * & & 0 & & & & * & & \\ {\tilde{x}}_{I11} & \cdots & * & \cdots & {\tilde{x}}_{IJ1} & .... & {\tilde{x}}_{I1k} & \cdots & * & \cdots & 0 & .... & {\tilde{x}}_{I1K} & \cdots & * & \cdots & {\tilde{x}}_{IJK} \end{array}\right]$$
  where the columns corresponding to the particular environment *j* for different attributes *k* (*k* = 1,…,*K*) was discarded.
3. The third option was a combination of independent elements in *I*×*K*(*J*-1) columns and adjusted dependent elements in (*K*−1) × *r*_*kk*′_ columns.
  $$\left[\begin{array}{ccccccccccccccccc} {\tilde{x}}_{111} & \cdots & {\tilde{x}}_{1j1}r_{1k(j)} & \cdots & {\tilde{x}}_{1J1} & .... & {\tilde{x}}_{11k} & \cdots & * & \cdots & 0 & .... & {\tilde{x}}_{11K} & \cdots & {\tilde{x}}_{1jK}r_{kK(j)} & \cdots & {\tilde{x}}_{1JK}\\ \vdots & & & & 0 & & \vdots & & * & & 0 & & & & 0\cdot r_{kK(j)} & & \\ 0 & & & & \vdots & & 0 & & * & & 0 & & & & \vdots & & \\ {\tilde{x}}_{I11} & \cdots & {\tilde{x}}_{Ij1}r_{1k(j)} & \cdots & {\tilde{x}}_{IJ1} & .... & {\tilde{x}}_{I1k} & \cdots & * & \cdots & 0 & .... & {\tilde{x}}_{I1K} & \cdots & {\tilde{x}}_{IjK}r_{kK(j)} & \cdots & {\tilde{x}}_{IJK} \end{array}\right]$$
  where the column corresponding to the particular environment *j* for particular attribute *k* was discarded, but the other columns which were discarded in the second option were replaced by those columns multiplied by their corresponding correlation coefficient between attribute *k* and *k*′(*k*′ = 1,…,*K*, *k*′≠*k*) for the same environment *j*, i.e. there were (*KJ*-1) columns in the design matrix. The correlation coefficients *r*_*kk*′(*j*)_ are described in the correlation matrix **R** (shown later).
4. The fourth option contained elements from the *I* genotype responses for the same environment over the (*K*-1) attributes, i.e. an *I*×(*K*-1) matrix.
  $$\left[\begin{array}{ccccccccccccccccc} {}* & * & {\tilde{x}}_{1j1} & * & * & .... & * & * & * & * & * & .... & * & * & {\tilde{x}}_{1jK} & * & *\\ {}* & * & & * & * & & * & * & * & * & * & & * & * & 0 & * & *\\ {}* & * & & * & * & & * & * & * & * & * & & * & * & \vdots & * & *\\ {}* & * & {\tilde{x}}_{Ij1} & * & * & .... & * & * & * & * & * & .... & * & * & {\tilde{x}}_{IjK} & * & * \end{array}\right]$$
  where the columns corresponding to the particular environment *j* with different attributes *k*′ (*k*′ = 1,…,*K*, *k*′≠*k*) were retained and all other columns were discarded.

The above design matrices could also be expressed as a partition into two matrices, one containing ($I-I_{jk}^{m}$) rows in $X_{obs}^{*}$, and the other containing $I_{jk}^{m}$ rows in $X_{m}^{*}$.

#### Estimation of missing cells

When the parameters **β**_*jk*_ and $\sigma_{jk}^{2}$ were determined (explained below), the missing cells ${\tilde{x}}_{jk}^{m}$ in one environment *j* for one attribute *k* could be drawn from Eq (2) [5]:

$${\hat{\tilde{x}}}_{jk}^{m}=X_{m}^{*}{\hat{\beta }}_{jk}+z{\hat{\sigma }}_{jk}$$ (2)

where **z** is random standard Normal vector, and $X_{m}^{*}$ is the corresponding design matrix described above. For the third way of determining the design matrix **X** ^*^, the correlation matrix *Cor*_*j*_ among attributes for any environment *j*, *j* = 1,…,*J* is:

$$Cor_{j}=\left[\begin{array}{ccccc} 1 & r_{12(j)} & \cdots & \cdots & \cdots \\ & 1 & \ddots & \ddots & \\ & & \ddots & r_{k^{\prime }(k^{\prime }+1)(j)} & \ddots \\ & & & \ddots & \ddots \end{array}\begin{array}{c} r_{1K(j)}\\ \vdots \\ \vdots \\ r_{\left(K-1)K(j\right)}\\ 1 \end{array}\right].$$

We took the distinct upper off-diagonal elements and wrote them as a *K*(*K*-1)/2 row vector $r_{j}^{\prime }=(r_{12(j)},\cdots r_{k^{\prime }(k^{\prime }+1)(j)}\ldots ,r_{\left(K-1)K(j\right)})$, where *k*′ = 1,…,*K*,. Then we combined all such vectors for each of the *J* environments to obtain a *J*×*K*(*K*-1)/2 full correlation matrix **R**, shown as follows:

$$R=\left[\begin{array}{c} r_{12(1)}\\ r_{12(2)}\\ \begin{array}{c} \begin{array}{c} \vdots \\ r_{12(j^{\prime })} \end{array}\\ \vdots \end{array}\\ r_{12(J)} \end{array}\begin{array}{c} \cdots \\ \vdots \\ \begin{array}{c} \begin{array}{c} \vdots \\ \vdots \end{array}\\ \vdots \end{array}\\ \vdots \end{array}\begin{array}{c} r_{1K(1)}\\ r_{1K(2)}\\ \begin{array}{c} \begin{array}{c} \vdots \\ r_{1K(j^{\prime })} \end{array}\\ \vdots \end{array}\\ r_{1K(J)} \end{array}\begin{array}{c} r_{23(1)}\\ r_{23(2)}\\ \begin{array}{c} \begin{array}{c} \vdots \\ r_{23(j^{\prime })} \end{array}\\ \vdots \end{array}\\ r_{23(J)} \end{array}\begin{array}{c} \cdots \\ \vdots \\ \begin{array}{c} \begin{array}{c} \vdots \\ \vdots \end{array}\\ \vdots \end{array}\\ \cdots \end{array}\begin{array}{c} r_{2K(1)}\\ r_{2K(2)}\\ \begin{array}{c} \begin{array}{c} \vdots \\ r_{2K(j^{\prime })} \end{array}\\ \vdots \end{array}\\ r_{2K(J)} \end{array}\begin{array}{c} \cdots \\ \vdots \\ \begin{array}{c} \begin{array}{c} \vdots \\ \vdots \end{array}\\ \vdots \end{array}\\ \cdots \end{array}\begin{array}{c} r_{k^{\prime }(k^{\prime }+1)(1)}\\ r_{k^{\prime }(k^{\prime }+1)(2)}\\ \begin{array}{c} \begin{array}{c} \vdots \\ NA \end{array}\\ \vdots \end{array}\\ r_{k^{\prime }(k^{\prime }+1)(J)} \end{array}\begin{array}{c} \cdots \\ \begin{array}{c} \begin{array}{c} \vdots \\ NA \end{array}\\ \vdots \end{array}\\ \cdots \end{array}\begin{array}{c} r_{k^{\prime }K(1)}\\ r_{k^{\prime }K(2)}\\ \begin{array}{c} \begin{array}{c} \vdots \\ NA \end{array}\\ \vdots \end{array}\\ r_{k^{\prime }K(J)} \end{array}\begin{array}{c} \ldots \\ \vdots \\ \begin{array}{c} \begin{array}{c} \vdots \\ \vdots \end{array}\\ \vdots \end{array}\\ \cdots \end{array}\begin{array}{c} r_{\left(K-1)K(1\right)}\\ r_{\left(K-1)K(2\right)}\\ \begin{array}{c} \begin{array}{c} \vdots \\ r_{\left(K-1)K(j^{\prime }\right)} \end{array}\\ \vdots \end{array}\\ r_{\left(K-1)K(J\right)} \end{array}\right],$$

where *NA* indicates that the correlation coefficients between attribute *k'* and the other attributes for environment *j*′ are not available (i.e. the genotype responses for environment *j*′ for attribute *k'* are missing).

#### Estimation of missing columns

When there is a missing column (${\tilde{x}}_{j^{\prime }k^{\prime }}^{m}$, an *I*×1 vector), there are no observations for that particular attribute in that particular environment. It is impossible to estimate parameters **β**_*j*′*k*′_ and $\sigma_{j^{\prime }k^{\prime }}^{2}$ from the non-missing values in this column (as there are no non-missing values). Therefore, we propose using the correlation coefficients to estimate the missing column.

However, as shown in the correlation matrix **R**, the correlation coefficients between attribute *k*′ and the other attributes for environment *j*′ were not available. Thus, the replacement of these correlation coefficients was computed by the average correlation (${\bar{r}}_{kk^{\prime }}=\frac{\sum_{j=1}^{J}r_{kk^{\prime }(j)}}{J-1}$, *j*≠*j*′) between attribute *k*′ and the other attributes over the (*J*-1) environments. Then, the average correlation between attribute *k*′ and the other attributes acted as the linear regression coefficient for this particular environment *j*′. Therefore, the particular missing column was estimated as follows:

$${\hat{\tilde{x}}}_{j^{\prime }k^{\prime }}^{m}=\frac{\sum_{k=1}^{K}{\bar{r}}_{kk^{\prime }}{\tilde{x}}_{j^{\prime }k}}{K-1}+z\bar{\hat{\sigma }},k\neq k^{\prime },$$ (3)

where $\bar{\hat{\sigma }}$ is the average value of the σ_*jk*_ from the other non-missing environments for each attribute (i.e. *K*(*J*-1)).

Alternatively, missing correlation coefficients in the matrix **R** could be considered as some of the elements in the response vector $r_{j^{\prime }}^{\prime }$ (*j*′ = 1,…,*J*, *j*′≠*j*) which is assumed to be linearly related to the respective correlation coefficients for the different environments *j* (*j* = 1,…,*J*, *j*≠*j*′) in **R**. That is equivalent to

$$r_{j^{\prime }}^{\prime }=\beta_{jj^{\prime }}r_{j}^{\prime }$$

where **β**_*jj*′_ could be obtained by the MLE, and then the (*K*-1) missing correlation coefficients (*r*_*k*′1(*j*′)_,…,*r*_*k*′*K*(*j*′)_, *k*′ = 1,…,*K*, *k*′≠*k*) were estimated by linear regression. Therefore, the particular missing column was estimated by following equation:

$${\hat{\tilde{x}}}_{j^{\prime }k^{\prime }}^{m}=\frac{\sum_{k=1,k\neq k^{\prime }}^{K}r_{j^{\prime }}^{\prime }{\tilde{x}}_{j^{\prime }k}}{K-1}+z\bar{\hat{\sigma }}.$$ (4)

Eqs (3) and (4) were used to estimate the values in the other missing columns.

We wanted to obtain *H* imputed values for the missing values, so the process of using Eqs (2), (3) and (4) was repeated *H* independent times.

Using the above process, the estimation of values in the missing columns (corresponding to a particular environment-attribute combination) is based on measurements of the observed attributes in the same environment and they are not independent of those other attributes measured on the genotypes in that environment. On the other hand, the estimation of values for single missing cells in particular columns is based on one of four ways of combining other observations, and the optimum combination is determined by the accuracy of estimation performance.

To obtain estimates of parameters **β**_jk_ and $\sigma_{jk}^{2}$ in the above, both Bayesian analysis and non-Bayesian analysis were used.

To conduct Bayesian analysis, we assumed that the prior distribution of **β**_jk_ was normally distributed with **β**_*jk*_ ∼ N(**β**_0_, **V**_*β*_) and $\sigma_{jk}^{2}$ was inverse-gamma distributed with $\sigma_{jk}^{2}∼\mathrm{IG}(\nu_{0}/2,S_{0}/2)$. We followed Gelman [18] in assuming that **V**_*β*_ = *τ* ^2^ **I**, and the hyper-parameters **β**_**0**_, *ν*_0_, *S*_0_ and *τ* ^2^ were fixed and known. As the sample variance of each observed column vector ${\tilde{x}}_{jk}^{obs}$ is 1, the shape parameter *ν*_0_/2 and scale parameter *S*_0_/2 have *ν*_0_ and *S*_0_ set to 4 and 2, respectively, Thus the mean of $\sigma_{jk}^{2}$ with inverse-gamma prior distribution is 1 ($=\frac{S_{0}/2}{\nu_{0}/2-1}$). In addition, the sample mean of each observed column vector ${\tilde{x}}_{jk}^{obs}$ is zero, hence, the mean of **β**_*jk*_ with normal prior distribution is **0** (= **β**_**0**_).

Thus, the full conditional distributions $f(\sigma_{jk}^{2}|{\tilde{x}}_{jk},\beta_{jk})$ and $f(\beta_{jk}|{\tilde{x}}_{jk},\sigma_{jk}^{2})$, are derived based on the Bayes’ rule as follows:

$$f(\sigma_{jk}^{2}|{\tilde{\text{x}}}_{jk},\beta_{jk})\propto f(\sigma_{jk}^{2},\beta_{jk}|{\tilde{\text{x}}}_{jk})\propto f({\tilde{\text{x}}}_{jk}|\beta_{jk},\sigma_{jk}^{2})f(\sigma_{jk}^{2})f(\beta_{jk}),$$

and

$$f(\beta_{jk}|{\tilde{x}}_{jk},\sigma_{jk}^{2})\propto f(\beta_{jk},\sigma_{jk}^{2}|{\tilde{x}}_{jk})\propto f({\tilde{x}}_{jk}|\beta_{jk},\sigma_{jk}^{2})f(\beta_{jk})f(\sigma_{jk}^{2})$$

where

$$f({\tilde{\text{x}}}_{jk}|\beta_{jk},\sigma_{jk}^{2})={\left(2\pi \right)}^{-I/2}|\sigma_{jk}^{2}\text{I}{|}^{-1/2}\exp(-\frac{1}{2}{\left({\tilde{\text{x}}}_{jk}-{\text{X}}^{*}\beta_{jk}\right)}^{T}{\left(\sigma_{jk}^{2}\text{I}\right)}^{-1}({\tilde{\text{x}}}_{jk}-{\text{X}}^{*}\beta_{jk})),$$

$$f(\sigma_{jk}^{2})\propto {\left(\sigma_{jk}^{2}\right)}^{-(\frac{\nu_{0}}{2}+1)}e^{-S_{0}/2\sigma_{jk}^{2}},$$

and

$$f(\beta_{jk})={\left(2\pi \right)}^{-(KJ-1)/2}|{\text{V}}_{\beta }{|}^{-1/2}\exp(-\frac{1}{2}{\left(\beta_{jk}-\beta_{0}\right)}^{T}{\left({\text{V}}_{\beta }\right)}^{-1}(\beta_{jk}-\beta_{0})).$$

Here, both $f(\sigma_{jk}^{2}|{\tilde{x}}_{jk},\beta_{jk})$ and $f(\beta_{jk}|{\tilde{x}}_{jk},\sigma_{jk}^{2})$ are proportional to the same product of distributions. Thus,

$$\begin{array}{c} f(\sigma_{jk}^{2}|{\tilde{x}}_{jk},\beta_{jk})\propto {\left(\sigma_{jk}^{2}\right)}^{-I/2}\exp(-\frac{1}{2\sigma_{jk}^{2}}{\left({\tilde{x}}_{jk}-X^{*}\beta_{jk}\right)}^{T}({\tilde{x}}_{jk}-X^{*}\beta_{jk})){\left(\sigma_{jk}^{2}\right)}^{-(\frac{\nu_{0}}{2}+1)}e^{-S_{0}/2\sigma_{jk}^{2}}\\ \propto {\left(\sigma_{jk}^{2}\right)}^{-(\frac{\nu_{0}+I}{2}+1)}\exp(-\frac{1}{2\sigma_{jk}^{2}}[{\left({\tilde{x}}_{jk}-X^{*}\beta_{jk}\right)}^{T}({\tilde{x}}_{jk}-X^{*}\beta_{jk})+S_{0}])\;,\\ \sigma_{jk}^{2}|{\tilde{x}}_{jk},\beta_{jk}∼\mathrm{IG}(\frac{\nu_{0}+I}{2},\frac{{\left({\tilde{x}}_{jk}-X^{*}\beta_{jk}\right)}^{T}({\tilde{x}}_{jk}-X^{*}\beta_{jk})+S_{0}}{2}), \end{array}$$

and

$$\begin{array}{l} f(\beta_{jk}|{\tilde{x}}_{jk},\sigma_{jk}^{2})\propto \exp[-\frac{1}{2\sigma_{jk}^{2}}{\left({\tilde{x}}_{jk}-X^{*}\beta_{jk}\right)}^{T}({\tilde{x}}_{jk}-X^{*}\beta_{jk})]\exp[-\frac{1}{2\tau^{2}}{\left(\beta_{jk}-\beta_{0}\right)}^{T}(\beta_{jk}-\beta_{0})]\\ \;\propto \exp\{-\frac{1}{2}[\beta_{jk}^{T}(\frac{{\left(X^{*}\right)}^{T}X^{*}}{\sigma_{jk}^{2}}+\frac{1}{\tau^{2}})\beta_{jk}\;\text{-}\;2(\frac{{\tilde{x}}_{jk}^{T}X^{*}}{\sigma_{jk}^{2}}+\frac{\beta_{0}^{T}}{\tau^{2}})\beta_{jk}]\}\\ \;∼N(A,B)\begin{array}{cccc} & & & . \end{array} \end{array}$$

Therefore, $B^{-1}=(\frac{{\left(X^{*}\right)}^{T}X^{*}}{\sigma_{jk}^{2}}+\frac{1}{\tau^{2}})$, and $A={\left(\frac{{\left(X^{*}\right)}^{T}X^{*}}{\sigma_{jk}^{2}}+\frac{1}{\tau^{2}}\right)}^{-1}(\frac{{\left(X^{*}\right)}^{T}{\tilde{x}}_{jk}}{\sigma_{jk}^{2}}+\frac{\beta_{0}}{\tau^{2}})$.

Thus, the full distribution $f(\beta_{jk},\sigma_{jk}^{2})$ can be obtained using Gibbs sampling.

From the above equations, when the prior distributions of parameters **β**_*jk*_ and $\sigma_{jk}^{2}$ were set up as the normal and inverse-gamma distribution, respectively, their corresponding conditional posterior distributions were also normal and inverse-gamma distribution, respectively. After the full distributions of parameters **β**_*jk*_ and $\sigma_{jk}^{2}$ were obtained, the estimates of **β**_*jk*_ and $\sigma_{jk}^{2}$ were obtained by generating a large number of extracted samples (here 5500) from those distributions and setting the estimate equal to their mean values, i.e.:

$${\hat{\beta }}_{jk}=mean(\beta_{jk})$$

$${\hat{\sigma }}_{jk}^{2}=mean(\sigma_{jk}^{2}).$$

Then the estimation of single missing values could be obtained by Eq (2) using the above estimates of parameters **β**_*jk*_ and $\sigma_{jk}^{2}$, and the estimation of a missing column could be obtained by Eqs (3) and (4).

Alternatively, to conduct the non-Bayesian analysis, we needed to obtain the MLEs for parameters **β**_*jk*_ and $\sigma_{jk}^{2}$. The log-likelihood function for ${\tilde{x}}_{jk}$ is:

$$l(\beta_{jk},\sigma_{jk}^{2};{\tilde{\text{x}}}_{jk})=-(I/2)\log(2\pi \sigma_{jk}^{2})-\frac{1}{2}\sum \frac{{\left({\tilde{\text{x}}}_{jk}-{\text{X}}^{*}\beta_{jk}\right)}^{T}({\tilde{\text{x}}}_{jk}-{\text{X}}^{*}\beta_{jk})}{\sigma_{jk}^{2}}.$$

Thus, the MLEs for parameters **β**_*jk*_ and $\sigma_{jk}^{2}$ are:

$${\hat{\beta }}_{jk}={\left({\left(X^{*}\right)}^{T}X^{*}\right)}^{-1}{\left(X^{*}\right)}^{T}{\tilde{x}}_{jk}$$

$${\hat{\sigma }}_{jk}^{2}=\frac{1}{I-2}\sum {\left({\tilde{x}}_{jk}-X^{*}{\hat{\beta }}_{jk}\right)}^{T}({\tilde{x}}_{jk}-X^{*}{\hat{\beta }}_{jk}).$$

Therefore, the estimation of single missing values could be obtained by Eq (2) using MLEs for parameters **β**_*jk*_ and $\sigma_{jk}^{2}$, and the estimation of a missing column could be obtained using Eqs (3) and (4).

### Predictive Mean Matching (PMM)

Rubin [12] proposed a statistical matching method for univariate nonresponse data while Little [13] developed and modified the method for multivariate nonresponse data, calling it predictive mean matching, where the respondent genotype vector (an *I*_0_×1 vector, *I*_0_<*I*) satisfied a regression model. The parameters in the model (i.e. the regression coefficients **β** and residual variance σ^2^) were determined by non-missing values. The predicted values of respondent genotype vectors, including non-missing and missing values, were obtained from the regression model. All predicted values for non-missing values were compared with the predicted values for the missing values by a distance function [13]. Then the *C* (*C*<<*I*_0_, e.g. 1, 2, and 3…, say 3) closest predicted values to a predicted missing observation implied that these particular *C* actual non-missing values could be used to estimate the missing value. One of these *C* values was chosen at random for each such missing value.

#### Estimation of missing cells

The regression model we used was again the normal regression model (NRM) as ${\tilde{x}}_{jk}∼N(X^{*}\beta_{jk},\sigma_{jk}^{2}I),\forall j,k$. Here, the design matrix employed one of the four options described above, i.e. the one determined to have the most accurate estimation performance. We drew a bootstrap sample of *I*_0_ observations (*I*_0_≤(*I*-*I*_*m*_)) from the non-missing (*I*-*I*_*m*_) values in the vector ${\tilde{x}}_{jk}^{obs}{}_{}$ (i.e. an (*I*-*I*_*m*_)×1 vector) for each imputation *h*, and put these values into a new vector ${\dot{\tilde{x}}}_{jk}$, which also contained the missing values, so it was of size (*I*_0_+*I*_*m*_). It contained two components, ${\dot{\tilde{x}}}_{jk}^{obs}$ of size *I*_0_×1, and ${\tilde{x}}_{jk}^{m}$ of size *I*_*m*_×1. The estimators of **β**_*jk*_ and $\sigma_{jk}^{2}$ were obtained using the same procedure as we described in the NRM imputation above but with a design matrix ${\dot{X}}^{*}$ which has (*I*_0_+*I*_*m*_) rows.

Each of the predicted values within the ${\hat{\dot{\tilde{x}}}}_{jk}^{obs}$ vector were compared with each of the predicted values within the ${\hat{\tilde{x}}}_{jk}^{m}$ vector using the following function [13]:

$$d_{(ii^{\prime })jk}^{2}={\left({\hat{\dot{\tilde{x}}}}_{ijk}^{obs}-{\hat{\tilde{x}}}_{i^{\prime }jk}^{m}\right)}^{2}\sigma^{2},i=1,\ldots ,I_{0},i^{\prime }=1,\ldots ,I_{m},\;\text{for each}\;j,k$$

where ${\hat{\dot{\tilde{x}}}}_{ijk}^{obs}$ are the elements within the ${\hat{\dot{\tilde{x}}}}_{jk}^{obs}$ vector, ${\hat{\tilde{x}}}_{i^{\prime }jk}^{m}$ are the elements within the ${\hat{\tilde{x}}}_{jk}^{m}$ vector. For each ${\hat{\tilde{x}}}_{i^{\prime }jk}^{m}$, $d_{(ii^{\prime })jk}^{2}$ (∀*i*′, *j*, *k*, *i* = 1,…,*I*_0_) has different *I*_0_ values, and we wanted to obtain the *C* smallest values from them. Then the estimate of each missing cell was randomly selected as one of these *C* corresponding actual values within the ${\dot{\tilde{x}}}_{jk}^{obs}$ vector for each imputation.

#### Estimation of missing columns

For a missing column (environment *j*′ in which attribute *k*′ was not measured), the predict function for this particular column is ${\hat{\tilde{x}}}_{j^{\prime }k^{\prime }}^{m}=\frac{\sum_{k=1}^{K}{\bar{r}}_{kk^{\prime }}{\tilde{x}}_{j^{\prime }k}}{K-1}$ or ${\hat{\tilde{x}}}_{j^{\prime }k^{\prime }}^{m}=\frac{\sum_{k=1}^{K}r_{kk^{\prime }(j^{\prime })}{\tilde{x}}_{j^{\prime }k}}{K-1}$, hence the predicted values of the observations in this missing column are related to the observations within the same environment *j*′ measured for the other attributes *k* (*k* = 1,…,*K*, *k*≠*k*′). Then the predict function for these observations (${\tilde{x}}_{j^{\prime }k}$) is ${\hat{\tilde{x}}}_{j^{\prime }k}=X_{I\times N_{k}(J-1)}^{*}\beta_{j^{\prime }k}^{*}$, where *N*_*k*_ is a randomly selected uniform sample from the *K* attributes, and $\beta_{j^{\prime }k}^{*}$ is a *N*_*k*_(*J*-1)×1 vector. The computation of estimators of parameters $\beta_{j^{\prime }k}^{*}$ and $\sigma_{j^{\prime }k}^{2}$ were the same as for the NRM imputation above using Gibbs sampling and MLEs.

As predicted values of observations within such a whole missing column were ${\hat{\tilde{x}}}_{j^{\prime }k^{\prime }}^{m}$, the distance $d_{ij^{\prime }(kk^{\prime })}^{2}$ was calculated between the elements of ${\hat{\tilde{x}}}_{j^{\prime }k^{\prime }}^{m}$ and ${\hat{\tilde{x}}}_{j^{\prime }k}$. Thus the estimate of each of the missing values ${\tilde{x}}_{j^{\prime }k^{\prime }}^{m}$ in the missing column was randomly drawn from one of the *C* closest corresponding actual values in ${\tilde{x}}_{j^{\prime }k}^{obs}$ (*k*⊰*N*_*k*_, *k*≠*k*′), where these *C* estimates were determined from the *C* smallest values of $d_{ij^{\prime }(kk^{\prime })}^{2}$.

### Comparison of methods

For each MI method discussed above, there were *H* imputed complete datasets, called “estimated data arrays”, containing the observed values in the incomplete data array and the imputed estimates of the missing values in that array. The overall or final “estimated data array” was obtained by averaging the cells in the *H* imputed data arrays. It could also be determined by averaging the *H* estimates of the individual missing values and putting them into the incomplete data array to form a complete array. Comparisons between the original data arrays and the “estimated data arrays” were conducted using the normal root mean square error (NRMSE) [19]

$$NRMSE=\sqrt{\frac{Mean[{\left({\tilde{x}}_{ijk}-{\hat{\tilde{x}}}_{ijk}\right)}^{2}]}{Var({\tilde{x}}_{ijk})}}$$

where ${\tilde{x}}_{ijk}$ is a standardized element in the original MET data array, and ${\hat{\tilde{x}}}_{ijk}$ is a standardized element in the “estimated data array”, as the standardized non-missing elements in the “estimated data array” are different from the standardized corresponding non-missing elements in the original MET data array. By considering each element of the data array in the NRMSE computation, we investigated both the influence of column standardisation prior to the analysis and the estimation performance. Also, the missing values could be estimated using the EM algorithm [9, 20, 21] in the three-mode ordination, referring to as the Tucker3 model [9, 22]. This method of estimating the missing values is defined as single imputation [9]. We therefore needed to compare the techniques we are proposing for estimating missing values (estimates for missing cells and estimates for a missing column) with those generated by the Tucker3 model. The NRMSE values for each MI method and the EM algorithm were compared to determine which method was more accurate for estimating missing values.

In the above, we considered both missing cells and missing columns simultaneously. However, we could consider the two patterns of missing values separately, i.e. missing cells alone and a missing column alone. We did that by comparing the estimation performance in each case using the following criterion to test the efficiency of multiple imputation.

There were *H* different imputed datasets for missing values in the full “estimated data arrays”. The corresponding imputed data values for the missing values, ${\hat{Q}}_{h}$, and variance of all missing values for each imputation *h*, *U*_*h*_, were obtained. For MI analysis, there are two types of variance [5, 6, 9, 23]. One is called the within-imputation variance and defined by $\bar{U}=\frac{1}{H}\sum_{h=1}^{H}U_{h}$. The other is referred to as the between-imputation variance and defined by $B=\frac{1}{\left(H-1\right)}\sum_{h=1}^{H}{\left({\hat{Q}}_{h}-\bar{Q}\right)}^{T}({\hat{Q}}_{h}-\bar{Q})$, where $\bar{Q}=\frac{1}{H}\sum_{h=1}^{H}{\hat{Q}}_{h}$. Then the total variance associated with the overall estimate is $T=\bar{U}+(1+\frac{1}{H})B$ [5].

Because we assessed these MI methods using complete data arrays from which we discarded values (those designated as “missing”), we know the original “true” or “actual” values of the missing values. Each element of the difference between the “true” values of missing values ***Q***_h_ and overall estimate $\bar{Q}$ divided by its overall standard deviation ($(Q_{h}-\bar{Q})/\sqrt{T}$) has an approximate *t* distribution with degrees of freedom $\nu_{H}=(H-1){\left[1+\frac{\bar{U}}{(1+H^{-1})B}\right]}^{2}$ [5, 24]. For such a *t* distribution, we could calculate the 95% confidence interval (CI) for the “actual” values, and then determine the percentage of these CIs which contained their corresponding “actual” values (of the total number of estimated values). This is referred to as Coverage CI [25]. The higher the value of Coverage CI, the more efficient the MI method is judged to be.

It was useful to repeat the MI process, as a different selection of cells would be designated as missing each time. We arbitrarily chose 10 repetitions and subsequently calculated a mean Coverage CI and standard error of that mean for each MI method.

MI approaches were considered in two ways, i.e. the same estimation procedure for missing cells and a missing column, and different estimation procedures for missing cells and a missing column. Thus, we divided our investigation of missing values for each repetition into missing cells alone, missing column alone, and combined (equivalent to all missing values). For missing cells alone and all missing values, we considered 5%, 10%, 15%, 20% and 25% missing values. This enabled us to evaluate the estimation performance of the MI approaches for each of these situations. For a missing column alone, we either deleted each environment for each attribute or deleted 10 distinct random environments for each attribute.

Firstly, the estimation procedure for missing cells alone was conducted for each MI approach, to give 100 imputed values for each missing cell. These 100 values were randomly allocated into 5 sets of 20 values, and the average over each of the 20 imputed values for each percentage of missing cells for each repetition gave the final 5 (*H*) imputed values for each missing cell. Based on those 5 imputed values for each missing cell, we calculated the 95% confidence interval for the “true” value. Then we obtained the percentage of all estimated missing cells where the 95% CI contained the “actual” value of the missing cell (Coverage CI). As the MI process was repeated 10 times, we calculated the mean Coverage CI and its standard error. Note that the percentage of missing cells for each repetition was slightly less than the percentage of missing values being quoted as there was a designated missing column (in the incomplete two-way wide array) whose cells were not included here.

Secondly, a missing column (corresponding to an attribute not measured on any genotype in a particular environment) was included in each percentage of missing values for each repetition. As it was randomly specified, it could be different across repetitions. We decided to investigate the variability of missing columns in the estimation procedure by carefully considering the choice of columns across replicates. For the small datasets (i.e. when the number of environments was 10 or less) each column was sequentially selected as the missing column (environment) for each attribute for each imputation in turn. The 100 imputed values of each missing data value in the missing column were obtained for each replicate. Again, 5 average imputed values (obtained by dividing the 100 values randomly into 5 sets of 20) of each missing value in the missing column were used to calculate the 95% Coverage CI. The repetitions gave *J* Coverage CIs (for each attribute) from which a mean and standard error were obtained. For the larger datasets (i.e. when the number of environments was greater than 10), we did not sequentially select each column (environment) to be missing in the replicates. Instead, we randomly selected 10 environments without replacement to be the missing column (environment) for each attribute for each imputation in turn. Then the same calculation was applied to obtain the 95% Coverage CI for each replicate. This gave 10 Coverage CIs (for each attribute) from which a mean and standard error were obtained.

Finally, we considered all missing values (missing cells and a missing column simultaneously). We obtained 100 imputed values of each missing value (whether it corresponded to a missing cell or to a cell in a missing column) for each percentage of missing values for each repetition of the MI process. To compare with the EM algorithm for estimating missing values, we took the average over the 100 imputed values of each missing value to form the final “estimated data array” for each percentage of missing values for each of 10 repetitions. We already had these final “estimated data arrays” for each of 10 replicates for the EM algorithm. Then the estimation performances of the different MI procedures and the EM algorithm were compared using the normal root mean squared error (NRMSE) criterion for each of the 10 replicates. These were presented as box plots for each method for each percentage of missing values.

### MET data arrays

We employed two real complete multivariate MET datasets and four simulated multivariate MET datasets. The first real multivariate MET dataset, described by Basford and Tukey [26], consisted of 58 soybean lines evaluated in 4 sites in Queensland Australia in each of 2 years (denoted as 8 environments) for 6 attributes, i.e. a 58×8×6 data array. The second was from the 2014 CIMMYT wheat breeding program. It contained 50 wheat lines grown in 31 environments with measurements on 4 attributes (i.e. a 50×31×4 data array). These were denoted as Datasets 1 and 2, respectively. The other four datasets were simulated to be of various sizes. They were based on other trials in the CIMMYT wheat breeding program, the first of size (60×10×6) and the second of size (80×15×6), denoted as Datasets 3 and 4, respectively, and maize trials from a commercial company, the third of size (100×20×5) and the fourth of size (120×60×4), denoted as Datasets 5 and 6, respectively.

For simulated MET datasets, we computed the variance components for each random effect (${\hat{\sigma }}_{G}^{2},{\hat{\sigma }}_{E}^{2},{\hat{\sigma }}_{GE}^{2},{\hat{\sigma }}_{\varepsilon }^{2}$) within each attribute from an analysis using the mixed linear model for three-way three-mode real MET datasets. The values of the variance components were estimated using restricted maximum likelihood [27, 28] as implemented in the ASREML software [29]. As the estimated variance components for genotype, environment, genotype×environment, and residuals were obtained, the data values were randomly multivariate normally distributed with mean zero and corresponding estimated variance-covariance matrix from the real multivariate MET data (with only genotypes having non-zero off-diagonal terms). Then these values were used to form the full simulated datasets.

## Results

The results for missing cells, a missing column, and overall missing values will be discussed in turn.

Firstly, Table 1 contains the mean and standard of Coverage CI (calculated over the 10 repetitions) for each MI estimation method for each percentage of randomly generated missing cells. Note that the percentage of missing cells is less than the percentage of missing values being quoted (because only missing cells were considered, not the missing column, i.e. the percentages in the table indicated the number of missing cells including those in the missing column).

**Table 1:** Mean and standard error (in brackets) of Coverage CI (%) for each percentage of missing cells estimated using MAHC, Bayesian analysis and non-Bayesian analysis of NORM, NRM and PMM methods, i.e. MAHC, NORM(BA), NORM(NBA), NRM(BA), NRM(NBA), PMM(BA) and PMM(NBA) for Datasets 1 to 6.

		MAHC	NORM(BA)	NORM(NBA)	NRM(BA)	NRM(NBA)	PMM(BA)	PMM(NBA)
**Dataset 1**	5%	86.5 (2.3)	82.5 (4.5)	82.5 (4.5)	81.6 (4.7)	81.6 (4.5)	82.7 (4.7)	82.6 (4.1)
	10%	84.0 (2.5)	79.7 (4.2)	79.2 (4.8)	79.6 (5.6)	79.6 (5.9)	79.7 (5.1)	79.7 (5.2)
	15%	80.8 (3.6)	76.1 (4.5)	76.7 (3.4)	76.5 (5.8)	76.5 (5.5)	76.5 (5.1)	76.4 (5.1)
	20%	78.1 (2.5)	74.5 (5.7)	73.8 (5.9)	73.3 (4.7)	73.4 (4.6)	73.6 (5.2)	73.5 (5.0)
	25%	76.9 (3.4)	72.9 (4.1)	71.9 (5.3)	72.5 (4.5)	72.6 (4.2)	72.9 (4.5)	72.7 (4.5)
**Dataset 2**	5%	84.1 (1.7)	82.4 (5.6)	81.9 (4.9)	81.7 (4.1)	80.3 (5.5)	82.0 (7.7)	81.9 (6.8)
	10%	81.8 (2.7)	80.1 (6.1)	79.2 (5.5)	78.8 (6.2)	77.8 (6.2)	79.4 (4.1)	79.6 (4.8)
	15%	78.7 (2.1)	76.1 (4.9)	75.4 (6.1)	75.2 (5.9)	74.9 (7.5)	75.7 (5.8)	75.2 (6.0)
	20%	76.0 (1.9)	73.8 (5.1)	72.9 (5.5)	71.2 (5.8)	70.9 (4.1)	72.9 (6.1)	72.8 (5.8)
	25%	74.2 (2.1)	71.9 (4.8)	70.2 (4.7)	69.4 (7.7)	68.7 (6.9)	70.2 (5.9)	70.0 (5.4)
**Dataset 3**	5%	84.0 (1.8)	79.1 (3.9)	79.0 (4.2)	76.6 (4.3)	76.6 (4.3)	78.5 (5.2)	77.7 (5.3)
	10%	82.8 (2.5)	76.1 (4.1)	75.8 (4.3)	72.2 (4.8)	72.2 (4.7)	76.6 (6.3)	76.3 (6.3)
	15%	79.6 (3.1)	73.9 (5.3)	73.5 (5.2)	71.7 (4.9)	71.6 (5.0)	72.9 (5.8)	72.1 (5.7)
	20%	76.9 (2.2)	72.0 (4.3)	71.5 (4.3)	69.2 (4.7)	69.0 (4.8)	70.6 (5.1)	70.5 (5.3)
	25%	72.9 (3.2)	69.7 (5.3)	69.5 (5.2)	65.2 (5.3)	65.1 (5.4)	68.3 (6.3)	68.3 (6.2)
**Dataset 4**	5%	82.3 (3.2)	79.8 (4.3)	79.7 (4.2)	77.2 (5.3)	77.1 (5.3)	78.8 (5.2)	78.0 (5.1)
	10%	80.0 (3.1)	78.0 (4.2)	77.6 (4.4)	75.8 (5.2)	75.6 (5.4)	77.4 (5.3)	77.2 (5.2)
	15%	78.5 (2.9)	75.8 (4.6)	75.6 (4.5)	72.4 (5.5)	72.0 (5.6)	75.0 (5.2)	74.6 (5.3)
	20%	75.5 (2.7)	72.1 (3.9)	72.0 (4.0)	69.7 (5.8)	69.2 (5.7)	70.4 (5.5)	70.2 (5.6)
	25%	72.6 (3.0)	71.0 (4.7)	70.8 (4.8)	66.9 (5.6)	66.7 (5.7)	69.7 (5.1)	69.5 (5.3)
**Dataset 5**	5%	85.0 (2.1)	80.9 (5.1)	80.5 (5.1)	79.2 (5.8)	79.1 (5.9)	80.2 (5.1)	80.1 (5.2)
	10%	81.7 (3.2)	78.1 (5.3)	78.1 (5.3)	77.9 (6.1)	77.8 (6.0)	78.6 (5.2)	78.5 (5.2)
	15%	80.0 (2.9)	77.0 (4.9)	76.6 (4.8)	75.4 (6.2)	75.3 (6.1)	76.0 (5.6)	75.9 (5.4)
	20%	76.6 (2.8)	74.2 (5.2)	74.1 (5.2)	71.6 (5.9)	71.3 (6.0)	73.6 (5.4)	73.6 (5.5)
	25%	73.1 (2.5)	71.1 (5.3)	71.0 (5.3)	69.6 (6.3)	69.5 (6.3)	70.5 (5.8)	70.4 (5.7)
**Dataset 6**	5%	83.3 (3.2)	79.8 (5.3)	79.8 (5.3)	77.7 (5.4)	77.6 (5.5)	78.4 (6.3)	78.2 (6.4)
	10%	80.8 (3.8)	77.0 (5.4)	76.6 (5.5)	74.6 (6.3)	74.2 (6.4)	75.8 (6.2)	75.4 (6.5)
	15%	77.1 (3.7)	74.2 (5.6)	74.1 (5.7)	72.3 (6.2)	72.0 (6.4)	74.0 (6.4)	73.7 (6.6)
	20%	74.2 (4.3)	70.5 (5.4)	70.3 (5.5)	69.7 (6.4)	69.6 (6.5)	70.9 (6.3)	70.3 (6.4)
	25%	71.3 (3.9)	68.7 (5.8)	68.0 (5.6)	66.6 (5.9)	66.1 (6.2)	68.0 (6.5)	67.4 (6.5)

As expected, the MAHC imputation was efficient, as the average coverage rates and their standard errors were good (means 74% or higher, standard errors less than 3.7%) for the real datasets (Datasets 1 and 2). For the simulated datasets (Datasets 3 to 6), the average coverage rates of MAHC imputation were similar to or slightly smaller than those for Datasets 1 to 2 (means over 71%), but the corresponding standard errors were somewhat higher (standard errors less than 4%) than those for the real datasets.

For the other imputation methods (NORM, NRM, and PMM) based on both analysis (Bayesian and non-Bayesian), the average coverage rates were lower and their standard errors were higher than those for MAHC at every percentage of missing cells for each dataset. Overall, both forms of NORM imputation had relatively higher coverage rates than those for the other two imputations, especially for larger datasets. The next best was PMM imputation.

On average, the results of Bayesian analysis of NORM imputations had slightly larger coverage rates than those for non-Bayesian analysis, but there was not much difference in coverage rates between Bayesian and non-Bayesian analysis for NRM and PMM imputations. The standard errors of the means for Bayesian and non-Bayesian analysis for the three imputation methods were quite similar at every percentage of missing cells. Also, the values of the standard errors of the coverage rates did not differ across the percentages of missing cells for most datasets.

Secondly, each environment was sequentially designated as the missing column for each attribute for the smaller datasets, while each of 10 random environments (chosen without replacement) was designated as the missing column for each attribute for the larger datasets. The mean and standard error of Coverage CI calculated over each environment missing in turn for each attribute for each estimation method for the smaller datasets and calculated over 10 environments (chosen at random without replacement) for each attribute for each estimation method for the larger datasets were presented in Table 2. The number of repetitions used to calculate average CI coverage for each dataset was 8 (for Dataset 1) and 10 (for Dataset 3), while the number of repetitions for Datasets 2, 4, 5 and 6 was 10. Therefore, Table 2 showed the average estimation performance for a missing column for each attribute for each dataset. The estimation procedures to estimate a missing column (environment) were the same as those to estimate missing cells for MAHC and both analysis of NORM imputation. For NRM and PMM imputations, we applied two relationships, “average” correlation coefficients and “linear” correlation coefficients, to estimate a missing column. The average Coverage CI of a missing column was expected to be similar across attributes for each dataset.

**Table 2:** Mean and standard error (in brackets) of Coverage CI (%) calculated over each environment missing in turn for each attribute (denoted as “A?”) for MAHC, Bayesian analysis and non-Bayesian analysis of NORM, average correlation and linear correlation of NRM and PMM methods, i.e. MAHC, NORM (BA), NORM (NBA), NRM (aver), NRM (linear), PMM (aver), and PMM (linear) for Datasets 1 to 6.

		MAHC	NORM(BA)	NORM(NBA)	NRM(aver)	NRM(linear)	PMM(aver)	PMM(linear)
**Dataset 1**	A1	80.6 (4.8)	78.7 (7.4)	78.7 (6.4)	78.2 (6.0)	77.3 (8.5)	78.2 (6.4)	77.3 (9.5)
	A2	84.7 (2.9)	82.7 (4.4)	82.0 (5.3)	79.5 (3.4)	79.3 (4.2)	79.3 (3.6)	79.1 (4.1)
	A3	84.1 (2.4)	80.7 (5.4)	80.3 (4.3)	79.5 (3.0)	79.4 (4.4)	79.5 (4.0)	79.3 (5.0)
	A4	84.9 (2.8)	84.6 (3.9)	83.6 (3.1)	81.0 (4.1)	80.5 (5.4)	80.9 (4.1)	80.4 (5.2)
	A5	84.1 (4.5)	80.8 (5.6)	80.4 (5.0)	78.4 (5.4)	77.9 (7.5)	78.3 (4.9)	77.3 (8.1)
	A6	84.2 (1.9)	84.0(2.5)	82.6 (3.8)	81.8 (3.1)	81.5 (5.9)	81.7 (3.2)	81.4 (6.7)
**Dataset 2**	A1	82.1 (2.7)	78.2 (7.7)	76.8 (6.8)	75.8 (7.4)	75.0 (7.6)	77.2 (6.9)	76.2 (4.6)
	A2	84.2 (2.9)	76.4 (8.4)	75.9 (5.8)	75.4 (8.4)	75.4 (8.2)	76.0 (6.2)	75.9 (7.1)
	A3	82.9 (1.9)	77.2 (5.5)	76.2 (5.9)	73.2 (5.8)	73.0 (4.7)	76.2 (5.9)	76.0 (6.0)
	A4	81.2 (2.9)	76.8 (5.5)	76.5 (6.9)	74.2 (6.0)	73.9 (5.9)	75.7 (4.7)	75.2 (4.5)
**Dataset 3**	A1	80.1 (1.2)	78.9 (4.3)	78.6 (5.1)	76.8 (1.7)	76.4 (2.9)	76.7 (1.4)	76.4 (1.8)
	A2	80.5 (0.9)	78.7 (4.7)	78.7 (5.1)	76.8 (1.0)	75.3 (1.7)	76.7 (1.2)	75.5 (1.1)
	A3	80.1 (1.1)	78.8 (4.4)	78.7 (5.5)	76.9 (1.2)	73.4 (2.1)	76.6 (1.1)	76.3 (1.4)
	A4	80.1 (1.2)	78.7 (3.9)	78.6 (4.3)	76.4 (1.3)	76.0 (3.2)	76.6 (1.1)	76.6 (1.4)
	A5	80.2 (1.0)	78.5 (5.1)	78.5 (5.2)	76.9 (1.3)	75.9 (4.0)	76.5 (1.7)	76.3 (1.6)
	A6	80.3 (1.2)	78.6 (4.9)	78.6 (5.2)	76.8 (3.6)	76.5 (3.5)	76.6 (1.4)	76.5 (1.2)
**Dataset 4**	A1	82.3 (0.8)	80.9 (2.6)	80.7 (4.0)	79.2 (1.3)	79.2 (1.5)	78.9 (1.1)	78.9 (1.1)
	A2	82.5 (1.1)	80.9 (1.6)	80.8 (2.2)	79.2 (2.2)	79.2 (3.2)	79.1 (5.8)	79.1 (7.1)
	A3	82.3 (1.5)	81.4 (2.2)	81.0 (3.1)	79.2 (2.8)	79.2 (4.2)	79.1 (6.1)	79.0 (6.5)
	A4	82.5 (2.2)	80.8 (3.5)	80.7 (2.7)	79.3 (3.5)	79.2 (5.1)	79.1 (2.7)	79.0 (3.5)
	A5	83.0 (2.8)	80.9 (4.4)	80.7 (5.8)	79.3 (2.4)	79.2 (3.3)	78.9 (4.8)	78.9 (5.9)
	A6	83.4 (3.1)	80.9 (1.1)	80.6 (4.2)	79.3 (4.4)	79.1 (5.8)	79.1 (3.7)	79.0 (4.2)
**Dataset 5**	A1	83.4 (2.2)	79.3 (4.2)	79.3 (2.3)	78.4 (2.8)	77.4 (3.6)	78.3 (4.8)	78.3 (5.1)
	A2	83.3 (1.8)	78.2 (5.5)	78.2 (3.7)	77.2 (4.3)	77.2 (6.1)	78.0 (2.3)	77.9 (3.6)
	A3	83.3 (3.4)	80.2 (1.2)	79.3 (1.1)	76.3 (3.8)	76.0 (4.2)	79.3 (1.5)	79.2 (1.7)
	A4	83.3 (1.6)	80.4 (3.5)	80.4 (4.2)	79.4 (1.7)	79.2 (2.8)	78.2 (2.7)	78.1 (3.2)
	A5	82.2 (2.3)	79.3 (2.7)	79.2 (4.8)	78.2 (2.4)	78.1 (5.1)	78.2 (4.4)	77.5 (4.8)
**Dataset 6**	A1	82.3 (2.3)	79.7 (2.5)	79.2 (3.2)	78.7 (1.2)	77.9 (2.1)	79.3 (2.9)	78.5 (3.8)
	A2	80.2 (1.4)	78.4 (1.8)	78.1 (1.2)	77.3 (2.3)	77.2 (3.8)	77.2 (3.3)	77.2 (4.2)
	A3	80.0 (1.3)	77.7 (3.5)	77.3 (1.7)	76.1 (3.9)	75.8 (4.5)	76.1 (1.5)	76.0 (1.8)
	A4	81.3 (2.8)	78.8 (1.1)	78.1 (2.8)	77.2 (1.9)	76.8 (2.5)	77.9 (4.4)	77.5 (5.6)

The MAHC imputation was very efficient as the average CI coverage rates were good (above 80%) for each dataset (Table 2). This was especially so for Dataset 1 where the average CI coverage rates were close to 85% for some attributes. In general, the average CI coverage rates for NORM imputation for both analysis were the next largest, followed by the other two imputations (NRM and PMM). The results of Bayesian analysis of NORM imputations had slightly larger average CI coverage values than those for non-Bayesian analysis. The standard errors of average CI coverage rates for MAHC imputation were smallest for a missing column for each attribute compared with other imputation methods. The standard errors for NORM imputation for Bayesian analysis were higher than those for non-Bayesian analysis for some attributes and lower for others. For NRM and NRM imputations, average correlation analysis had larger average CI coverage values and the lower standard errors than those from linear correlation analysis.

In general, the average coverage rates for both analysis of NORM imputation were relatively larger than those for NRM and PMM imputations. Again, the results of Bayesian analysis of NORM imputations had slightly larger average coverage rates than those from non-Bayesian analysis. The results of average correlation analysis of NRM and PMM imputations also had slightly larger average coverage rates and lower standard errors than those from linear correlation analysis.

Using the linear correlation analysis to calculate the missing column had slightly lower coverage rates than using the average correlation analysis (Table 2). Therefore, we subsequently used the average correlation analysis to compute the missing column for NRM and PMM imputation methods, in conjunction with Bayesian and non-Bayesian analysis to estimate missing cells for these two methods.

**Fig 3:**
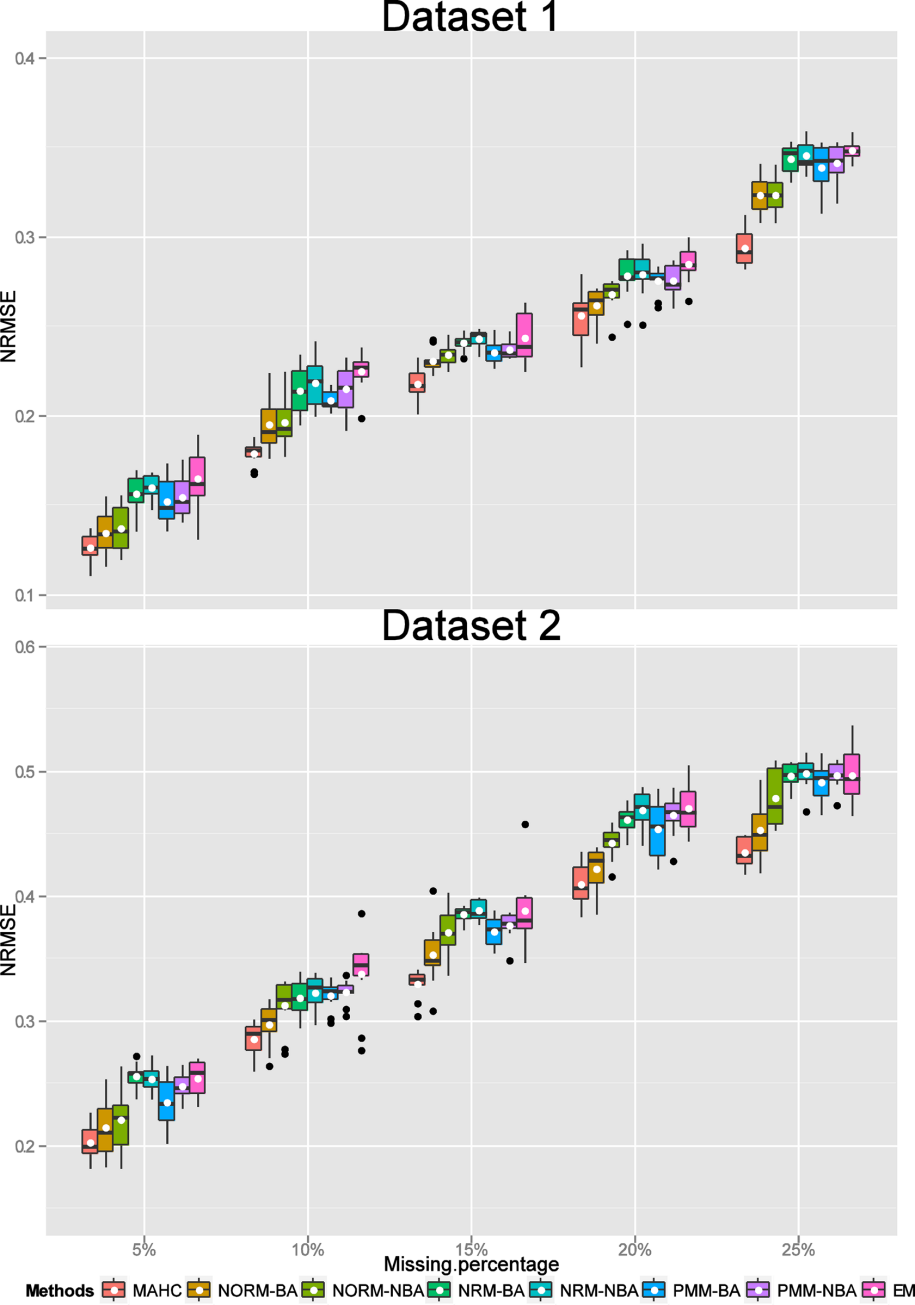
Boxplots of NRMSE values (with their mean denoted by a “white” dot). Comparison of the estimated missing values (including missing cells and a missing column) using MAHC, NORM-BA, NORM-NBA, NRM-BA, NRM-NBA, PMM-BA, PMM-NBA and EM methods with the “true” values for Datasets 1 and 2 for each percentage (5%, 10%, 15%, 20% and 25%) of missing data.

**Fig 4:**
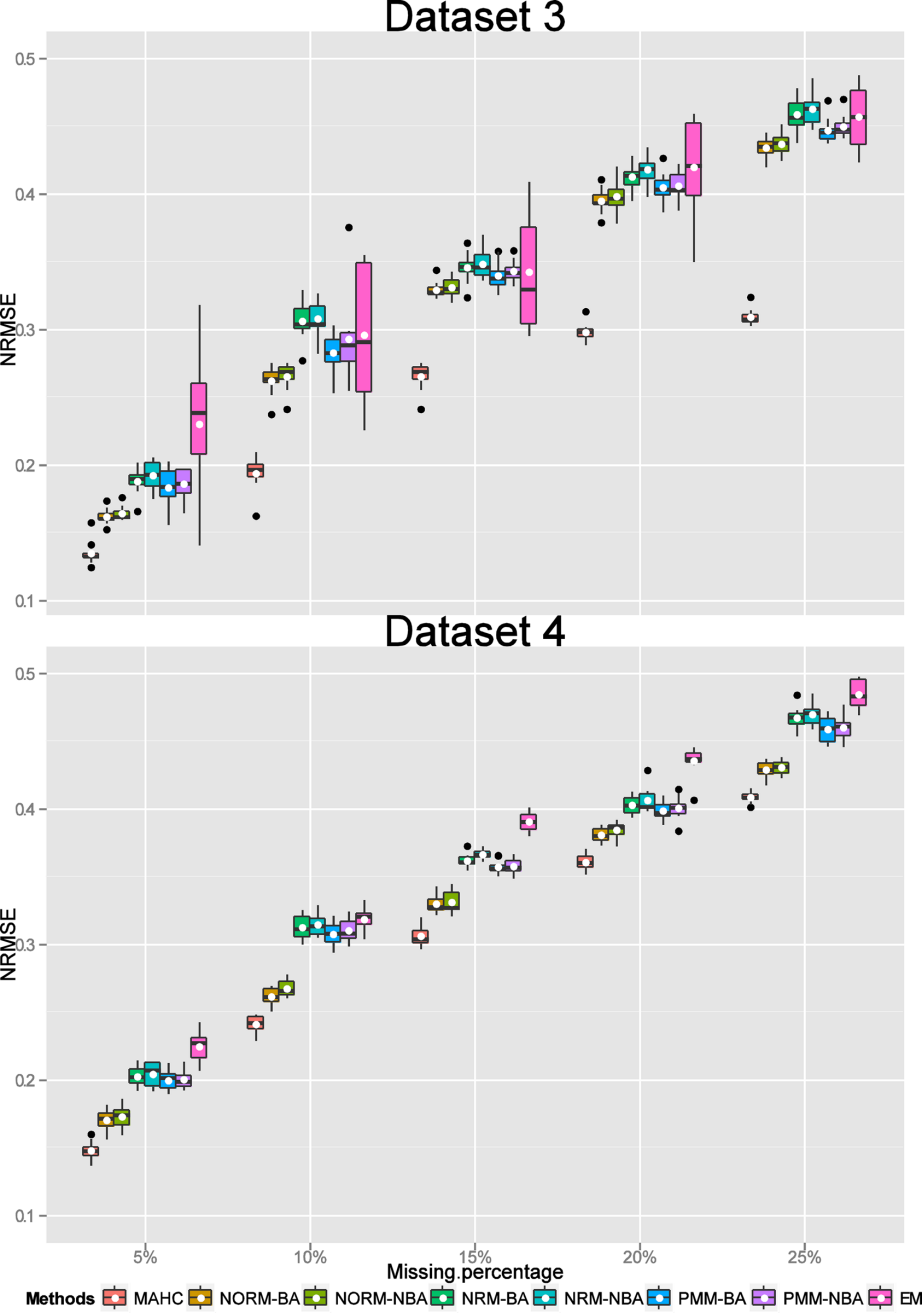
Boxplots of NRMSE values (with their mean denoted by a “white” dot). Comparison of the estimated missing values (including missing cells and a missing column) using MAHC, NORM-BA, NORM-NBA, NRM-BA, NRM-NBA, PMM-BA, PMM-NBA and EM methods with the “true” values for Datasets 3 and 4 for each percentage (5%, 10%, 15%, 20% and 25%) of missing data.

**Fig 5:**
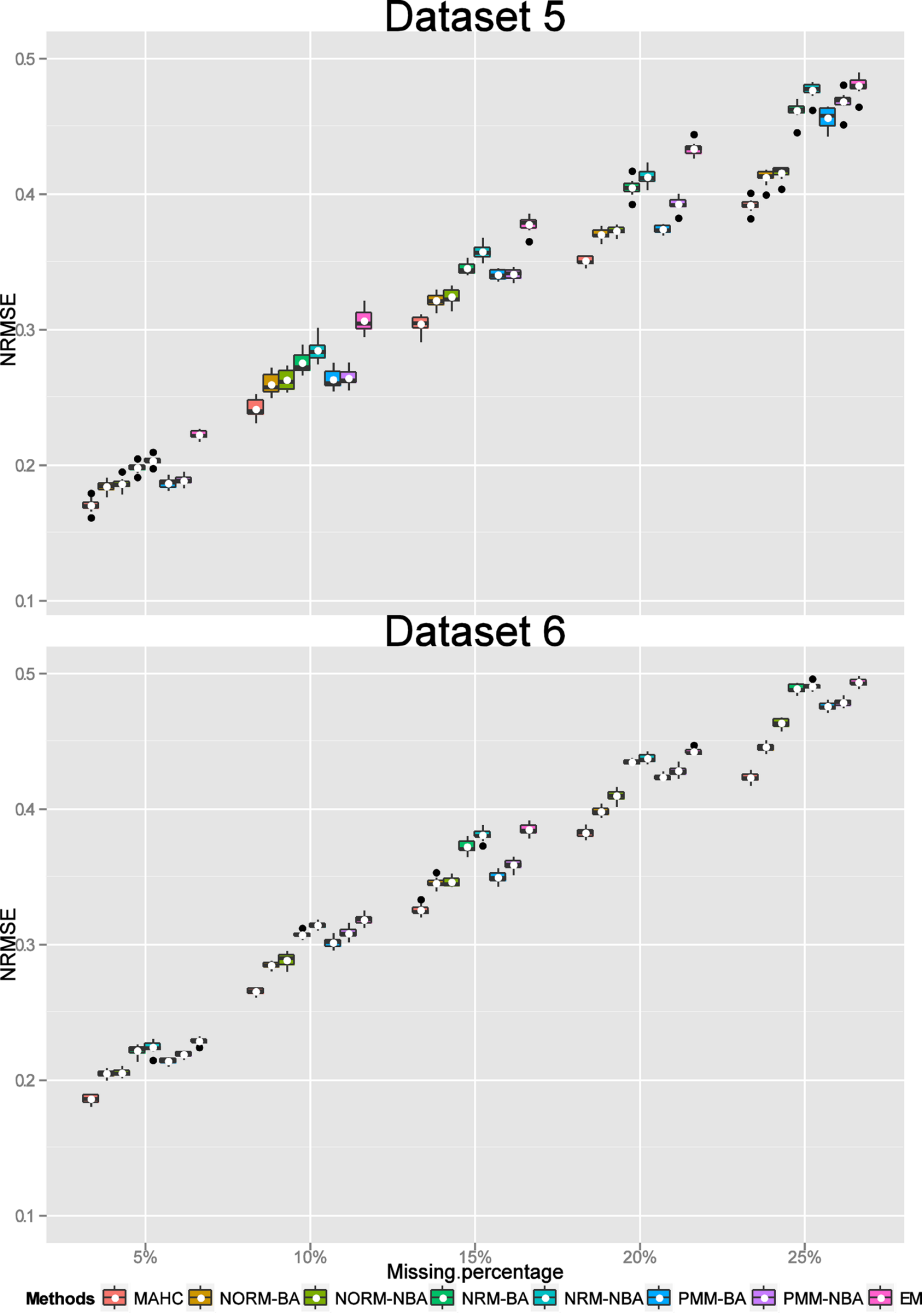
Boxplots of NRMSE values (with their mean denoted by a “white” dot). Comparison of the estimated missing values (including missing cells and a missing column) using MAHC, NORM-BA, NORM-NBA, NRM-BA, NRM-NBA, PMM-BA, PMM-NBA and EM methods with the “true” values for Datasets 5 and 6 for each percentage (5%, 10%, 15%, 20% and 25%) of missing data.

Consequently for missing values overall, we compared MAHC, NORM imputation with Bayesian and non-Bayesian analysis, NRM and PMM imputation with Bayesian and non-Bayesian analysis where the estimation of a missing column was via the average correlation analysis. For each MI approach and the EM algorithm for each percentage of missing values (including missing cells and a missing column), we obtained final estimated data values (by averaging over the 100 imputed estimates for each missing value) for each of 10 repetitions. The NRMSE values for assessing the accuracy of the MI approaches and the EM method for estimating missing values (by calculating the differences between the original “true” values and the estimated values) for each of the 10 replications for each percentage of missing values for each of Datasets 1 to 6 were displayed using a boxplot with the addition of the mean value (Figs 3–5). Low values of NRMSE correspond to better performance.

For Datasets 1 and 2 (the real datasets), the values of NRMSE for estimating the performance of EM, NRM-NBA, and NRM-BA imputation were comparably larger than those for the other imputations at every percentage of missing values (Fig 3). Overall, MAHC, NORM-BA and NORM-NBA imputations had relatively lower values of NRMSE, so performed better.

For Datasets 3 and 4 (of size 60×10×6 and 80×15×6, respectively), the values of NRMSE for estimating the performance of EM, NRM-NBA, and NRM-BA imputations were relatively higher than those for the other methods (Fig 4). Generally, MAHC imputation had much better performance than the other imputation methods for these two datasets. The variability of NRMSE values for EM imputation for Dataset 3 was much larger than for the other imputation methods. Overall, the variability of NRMSE values for Dataset 4 was smaller than for Dataset 3.

For Datasets 5 and 6 (of size 100×20×5 and 120×60×4, respectively), the values of NRMSE for estimating the performance of EM, NRM-NBA, and NRM-BA imputations were relatively higher than those for the other imputations (Fig 5). MAHC imputation had much better performance than the other imputation methods. The variability of NRMSE for these two datasets was much smaller than for the other datasets.

The overall best performance for estimating randomly generated missing values (including both missing cells and a missing column) for these six datasets was MAHC imputation, followed by NORM imputation for both Bayesian and non-Bayesian analysis. The number of missing values in relation to the size of the data array had an impact on the accuracy of estimating performance, with larger values of NRSME corresponding to larger percentages of missing data. However, the variability of the NRMSE values was smaller when the size of the dataset increased.

## Discussion

We investigated one multiple hierarchical clustering method (MAHC imputation) and three multiple imputation approaches (NORM, NRM and PMM) with and without Bayesian analysis for estimating missing values in three-way three-mode MET datasets.

To conduct Bayesian analysis using Gibbs sampling, we needed to assume some prior distributions for the parameters in the normal distribution model (NORM) and normal regression model (NRM). Also, we needed to set up some hyper-parameters in the prior distribution for NRM imputation. Thus, compared with NORM imputation, NRM imputation introduced more uncertainty and produced lower CI coverage rates and higher average values of NRMSE.

For the PMM imputation with and without Bayesian analysis, the estimated missing values were actual non-missing values from another cell plus a random standard normal term multiplied by an appropriate variance, where these actual non-missing values were those for which their predicted values were closest to the predicted missing values. The predicted values of non-missing values were obtained using the NRM model. The closest distance between predicted values of each cell based on the NRM model may differ from the closest distance between actual values of each cell. When that is the case, PMM imputation had lower coverage rates and higher average values of NRMSE than NORM imputation.

During the implementation of MAHC imputation, we employed different combinations of attributes for each imputation. Thus, the final estimated values using MAHC imputation were based on the average of the corresponding values from the same genotype in the environment (or environment group) deemed most like that particular environment when the “similarity” assessment used different combinations of attributes. It took advantage of multivariate measurements by combining the attributes in various ways in the estimation procedure. Another advantage was that there was no other uncertainty in MAHC imputation. The three other imputation approaches required the estimation of parameters in their underlying models. As a result, it is probably not surprising that MAHC imputation performed best.

For the three imputation models (NORM, NRM, and PMM), imputations with Bayesian analysis had slightly higher accuracy than those corresponding imputation models without Bayesian analysis, but they took more computer time to implement. For all comparisons with these imputations, MAHC imputation had the smallest implementation time and the highest accuracy.

For estimating a missing column, we made two different assumptions for the correlation among attributes for each environment. The correlation coefficients for the missing environment for a particular attribute were not available. Therefore, we decided that the missing correlation coefficients could be the average value of non-missing correlation coefficients or a linear combination of non-missing correlation coefficients. However, according to the results of our investigation, the first assumption (based on the average correlation coefficients) had better estimation performance than the second assumption (based on a linear combination of correlation coefficients). The linear combination of correlation coefficients was calculated using all other non-missing correlation coefficients among the attributes, while the average value of correlation coefficients was calculated from other non-missing correlation coefficients with this particular attribute. This is likely to be the reason that the average value introduced less uncertainty and produced higher CI coverage.

Multivariate multi-environment trial datasets are the focus of our research. As many of these are incomplete, it is important to estimate the missing values to form a complete array that can then be analysed by three-way pattern analysis methodology which has proven valuable for that situation. However, the above imputation methods could apply to other types of datasets where various measurements are made on the same entities under different conditions.

## Supporting Information

S1 Datasets Datasets used in this study. This compressed file contains six directories for each of six datasets. A description of each text file is included in the corresponding directory.
(ZIP)

S1 File R source code for multiple imputation approaches. (ZIP)
